# Age and gender-related neurophysiological changes in sleep and wake states during childhood

**DOI:** 10.1016/j.dcn.2026.101681

**Published:** 2026-01-21

**Authors:** Kevin Mammeri, Guillaume Legendre, Fiona Journal, Nathalie Fernandez, Helene Ruppen-Maret, Joanny Combey, Sophie Schwartz, Virginie Sterpenich

**Affiliations:** a Department of Basic Neurosciences, University of Geneva, Switzerland; b Swiss Center for Affective Science, Geneva, Switzerland

**Keywords:** EEG, Gender, Sleep, Aperiodic signal, Children

## Abstract

Brain maturation and sleep patterns evolve throughout childhood, intricately influencing cognitive functions. However, it remains unclear whether changes in sleep and cognition follow similar or distinct trajectories as a function of age and gender during childhood. We included 61 healthy children (30 boys and 31 girls), aged 5–12 years old, who completed a visual memory task and a sustained attention to response task (SART), before and after undergoing one night of polysomnography at home. Our findings revealed large age-related associations in girls, with N3 and REM durations decreasing and sleep spindle frequency during N2 increasing across development. Conversely, these patterns were not observed in boys. Moreover, a significant interaction showed a shift in delta power topography from posterior to anterior regions in girls compared to boys. Alongside these sleep changes, girls exhibited a predominant excitatory pattern of brain activity during wakefulness as evidenced by a reduction in resting EEG slope. Regarding cognition, we found a large correlation between the increase in sleep spindle frequency in girls and morning accuracy on the SART. Overnight memory consolidation did not vary with age or gender. Taken together, these findings suggest an earlier onset of brain maturation in girls, reflected by less deep sleep, faster sleep spindles, frontal shift in delta power, and greater cortical excitability during wakefulness. This pattern in girls challenges the notion that developmental modifications of sleep are minimal during childhood. How closely may these changes relate to puberty or timing remain to be established in future longitudinal studies.

## Highlights

• Brain activity was tracked at home to assess natural sleep and development.
• Girls show earlier reductions in deep and REM sleep than boys, reflecting brain development.
• Delta power shifts frontally only in girls, indicating faster brain maturation.
• Sleep spindle frequency rises with age in girls and predicts better attention accuracy.
• Gender specific brain changes during sleep matter for puberty, cognition, and mental health.

## 1 Introduction

During the first years of life, the human brain undergoes continuous maturation, shaping both neural structures and cognitive functions. Such brain maturation processes are closely and bidirectionally intertwined with changes in sleep physiology, which evolves from birth to adulthood (Buchmann et al., 2011, Kurz et al., 2023). From infancy to adolescence, total sleep duration gradually decreases, with a reduction in deep sleep (slow wave sleep, SWS or N3) and REM sleep, while lighter sleep stages (N1 and N2) increase in proportion (Agostini and Centofanti, 2021, Ohayon et al., 2004). Sleep architecture changes most rapidly in newborns, infants, and preschool-aged children, followed by a relatively stable period in school-aged children (5–12 years), before a steep decline in SWS is observed in teenagers (Barel and Tzischinsky, 2022). More specifically, SWS follows a dynamic inverted U-shaped time course as SWS is rising during early childhood, reaching a plateau between 5 and 12 years old, and decreasing during adolescence (Baker et al., 2012, Campbell and Feinberg, 2009, Tarokh et al., 2011, Jenni et al., 2005, Ringli and Huber, 2011). Sleep evolves not only at the macrostructural level but also through specific oscillatory patterns that fosters neural reorganization (Buchmann et al., 2011, Ringli and Huber, 2011). In particular, the posterior-to-anterior shift in slow-wave activity (SWA; 0.5–4 Hz) topography, observed between ages 5 and 14, mirrors the pattern of cortical grey matter maturation (Ringli and Huber, 2011, Kurth et al., 2012, Kurth et al., 2010, Riggins et al., 2024). This finding is in line with the hypothesis that slow waves may play an active role in synaptic pruning, especially during late childhood in prefrontal regions (Buchmann et al., 2011, Kurth et al., 2012, Feinberg et al., 2006). Interestingly, while SWS was found to correlate with age, it did not appear to be associated to other key biological markers of development such as height, weight, BMI, or sexual maturation (Feinberg et al., 2006). This intriguing observation hints at the possibility that changes in SWA may be a highly specific indicator of brain maturation, rather than of general physiological development (Ringli and Huber, 2011). Accordingly, both longitudinal and cross-sectional studies have highlighted the predictive value of sleep for the maturation of brain morphology. For instance, greater frontal SWS has been shown to predict higher global brain myelination three years later (LeBourgeois et al., 2019), while regular napping in 4- to 6-year-old children has been associated with a larger hippocampal body (Riggins and Spencer, 2020). Conversely, the maturation of hippocampal networks in children has been proposed to result in more efficient memory processes, yielding reduced sleep homeostatic pressure, thereby facilitating the transition away from napping (Riggins et al., 2024, Spencer and Riggins, 2022). These studies highlight the key bidirectional relationship between sleep and brain maturation.

Sleep spindles - a second key feature of NREM sleep – have also been shown to evolve during childhood and suggested to reflect brain maturation (Zhang et al., 2021, Gorgoni et al., 2020, Reynolds et al., 2018). Generated by thalamocortical networks, sleep spindles are defined by features such as density, frequency, duration, and amplitude, which show distinct trajectories with age (Purcell et al., 2017). For instance, spindle frequency (up to ∼13.5 Hz) increases linearly between 2 and 18 years of age, whereas spindle density follows an inverted U-shaped trajectory, similar to SWS (Zhang et al., 2021, Kwon et al., 2023). The concurrent decrease in spindle amplitude and duration, together with the increase in fast spindle density and frequency in frontal regions, all serve as reliable markers of brain maturation (Zhang et al., 2021, Purcell et al., 2017, Kwon et al., 2023, Goldstone et al., 2019, Ricci et al., 2021, Hahn et al., 2019). Age-related changes in sleep oscillatory features—slow waves and spindles—may thus be used as indexes of brain development.

Recent theoretical and technical advances in EEG research have refined this perspective by distinguishing the periodic (oscillatory) from the aperiodic (1/f-like) components of neural activity (Donoghue et al., 2020, Brake et al., 2024). In particular, this1/f-like curve, accounts for most of the signal and corresponds to the “aperiodic background”, while the peaks represent “periodic” or oscillatory activities (Donoghue et al., 2020). Critically, the aperiodic component was found to directly relate to global levels of neuronal activity (offset of the component) (Miller et al., 2014) and to synaptic and network properties, in particular excitation/inhibition balance across neuronal circuits (slope of the component) (Brake et al., 2024, Gao et al., 2017). A substantial body of literature indicates that the slope of the aperiodic component during wakefulness decreases with brain maturation (Cellier et al., 2021, McKeon et al., 2024), suggesting that excitatory activity in wakefulness increases as one gets older. Therefore, both periodic and aperiodic EEG components offer complementary windows onto the neurophysiology of sleep and its interaction with brain maturation.

Importantly, brain development does not follow the same trajectory in boys and girls. Gender differences in brain volume, cortical thickness, and gyrification are already emerging by the second year of life (Li et al., 2014). Beyond the brain, differences in the timeline of sexual maturation during development lead to earlier changes in morphological sexual characteristics (breast, genitals, pubic hair), reproductive capability, height, weight, and shift in chronotype in girls compared to boys (Alotaibi, 2019). These developmental timing differences make it plausible that sleep physiology may also follow distinct maturational trajectories across genders. However, most studies on sleep have not compared genders directly, except for instance, Himanen et al. (Himanen et al., 2017) who investigated gender differences in SWA in 28 healthy children aged 7–11 years. Their results showed no differences between genders, consistent with the idea of a stable period of preschool-aged children. Similarly, Feinberg et al. (Feinberg et al., 2006) showed no gender-related difference in delta power in 9–11 years-old children. In contrast, several studies report that such differences emerge during adolescence, with girls aged 12 and older showing greater SWS in frontal regions and higher sleep spindle frequencies compared to boys (Feinberg et al., 2006, Goldstone et al., 2019, Ricci et al., 2021, Markovic et al., 2020). Also, less mature adolescents (mostly boys) showed larger changes in spindle density compared to more mature adolescents (mainly girls), indicating that thalamocortical maturation supporting the generation of sleep spindles occur earlier in girls (Ricci et al., 2021, Colrain and Baker, 2011). The underlying mechanism for these gender differences is posited to stem from the earlier synaptic pruning in thalamocortical networks and frontal grey matter associated with an earlier onset of puberty in girls (Zhang et al., 2021, Ricci et al., 2021, Giedd, 2004, Ringli et al., 2013). However, periodic and aperiodic modifications during childhood have rarely been studied from a gender-dependent perspective.

Aside from their potential developmental contribution, periodic and aperiodic components have been associated with cognitive functions. Indeed, although literature on the beneficial effects of sleep on memory in children remains inconclusive, especially regarding sleep duration (Astill et al., 2012, Wilhelm et al., 2012), sleep spindles and SWS have at multiple times been linked to better memory and executive functions in children (Riggins and Spencer, 2020, Gorgoni et al., 2020, Hahn et al., 2019, Morales-Ghinaglia et al., July 2025, Mason et al., 2021). Sleep spindles, for instance, create permissive conditions for synaptic modifications and the initiation of long-term potentiation (Chatburn et al., 2013, Fogel and Smith, 2011, Cowan et al., 2020). While sleep spindles likely play a crucial role in memory consolidation, they have also been broadly linked to intelligence and executive functions (Geiger et al., 2011). Specifically, Chatburn et al. (Chatburn et al., 2013) reported that sleep spindle frequencies are associated with executive functions such as working memory and planning in healthy children. These cognitive improvements are thought to result from the maturation of brain regions involved in memory and executive functions. For instance, hippocampal subfield volumes differ between 4- to 6-year-old habitual and non-habitual nappers, suggesting more efficient memory storage in children who no longer nap (Riggins and Spencer, 2020). As previously described, the maturation of prefrontal cortical regions, which underlies the development of executive functions (Paus, 2005, Pöpplau and Hanganu-Opatz, 2024), is linked to the developmental shift of SWS towards prefrontal regions (Kurth et al., 2012, Kurth et al., 2017, Ong et al., 2022).

Although the relationship between sleep and brain maturation during adolescence has been extensively studied, there is a scarcity of research focused on these patterns during the school-age period. Moreover, the different trajectory of development between boys and girls is also not well-known. This study aims to **(i)** describe changes in brain activity and excitability during sleep and wakefulness in childhood, as recorded by EEG (**ii)** explore the association between sleep characteristics and cognitive performance during wakefulness, (**iii**) report any gender-related differences in sleep and/or cognitive developmental trajectories.

## 2 Method

### 2.1 Participants

A total of 61 healthy children from 5 to 12 years old (*M* = 8.0 years old, *SD* = 1.7, 31 girls) were recruited in the Geneva area (in Switzerland and France). No neurological, psychological or sleep disorders were reported by the parents at screening. Pubertal status was not examined as we chose an ecological setting at home without any medical specific examination. All children had French as mother tongue. The study was conducted with the approval of the Geneva University Research Ethics Commission (CUREG), and in accordance with the Declaration of Helsinki.

### 2.2 Procedure

**Fig. 1:**
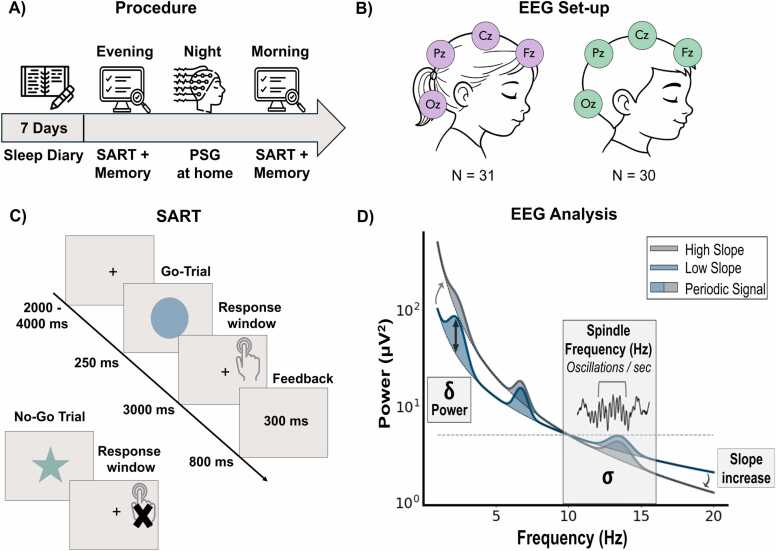
Experimental Procedure and Statistical EEG Approach. (A) Timeline of the study. (B) Representation of the EEG set-up composed of 4 electrodes in girls and boys. (C) Schema of one go and one no-go trial in the SART task. (D) Representation of the different EEG features extracted: periodic (delta power and spindle frequency) and aperiodic (slope) components. PSG: polysomnography.

A first visit at the child’s home was organized to inform the family about the specifics of the study, and to ensure that the child was healthy and willing to participate. Parents had to sign an ethical consent form. For one week, parents with their child had to fill in a diary form (adapted for children) assessing daily mood, sleep and dreams. During the evening of the seventh day, the experimenter came to the child’s home for a first cognitive session including a visual memory task and a sustained attention to response task (SART). A mobile EEG was set-up for the whole night. The subjects slept at home in their own beds following their usual school-night sleep schedules, without any supervision of the experimenter. The experimenters came back the next morning to remove the EEG set up and administer the memory task and SART again (see Fig. 1A).

### 2.3 Subjective sleep measures

Subjective measures such as bedtime, sleep onset (defined as the time at which participants fell asleep), waking-up time (defined as the time at which participants woke up in the morning) and sleep quality were collected via the sleep diary. We then calculated the difference between sleep onset and waking-up time to obtain the total sleep time (TST) in hours, which represents a subjective measure of sleep duration during the night. An average over the seven days was computed to obtain a measure of subjective sleep duration.

### 2.4 EEG data acquisition

Polysomnographic recordings were performed using a small mobile EEG system (Avatar amplifier, Magstim Electrical Geodesics, Inc. (EGI), Oregon, USA) with 6 EEG electrodes (Oz, Pz, Cz, Fz, A1 and A2 (placed on the mastoids) according to the international 10–20 system), 2 EOG electrodes and 2 EMG electrodes (see Fig. 1B). The mastoids’ location was used for reference electrodes. Impedances of all the electrodes were kept below 5 kΩ and the sampling rate used for the recording was fixed at 500 Hz.

### 2.5 Cognitive tasks

An image-location memory task adapted from Rasch et al. (Rasch et al., 2007) was modified for children to explore episodic memory consolidation during sleep. During the evening session, children encoded two grids, each with 5 × 4 cells containing 10 pairs of images (5 pairs of candies and 5 of vegetables) distributed at random cell-locations on the grids. Each pair was displayed for 2 s on a grid, and children had to memorize the spatial location of each image of the pair. A first retrieval occurred just after encoding, during which children were shown, in a random and different order, one image from a pair and asked to recall the location of the second similar image. Feedback was provided after each trial. If a child's score was below 5 out of 10, the encoding and recall were repeated once more, for a maximum of two encoding attempts. Children were informed that correctly recalling candy pairs would be rewarded with real candies the next morning, while vegetable pairs would not result in a reward. During the morning session, children recalled the image pairs, presented in a random and different order from the evening session. The task aimed to investigate the role of sleep in memory consolidation in children, using stimuli with high (candies) and low (vegetables) reward values. Recall accuracy for both rewarded and non-rewarded images was investigated for the evening and the following morning tests.

A child-adapted SART was assessed during the evening and morning sessions. The task involved the random presentation of 10 different geometric forms in various colours, with instructions to press a key as soon as a form appeared on the screen, but to refrain from responding when a green star was displayed. Each trial began with a fixation cross shown for 2–4 s, followed by the geometric form presented for 250 ms. Participants then had 3 s to respond, after which feedback on their reaction time was displayed for 0.8 s before the next trial began (see Fig. 1C). Mean reaction times and accuracy were used as outcome measures. Accuracy was defined as the difference between the proportion of correct responses to go trials and the proportion of incorrect responses to no-go trials. Scores ranged from 0 (worst performance) to 1 (best performance). To uncover possible links between the preceding night’s sleep and performance on the SART, we focused on the results from the morning session.

These two tasks were performed in the evening at around 7.03 p.m. (± 55 min) (between 6–8 p.m.) and lasted 51 ± 13 min and in the morning at around 9.18 a.m. (± 43 min) (between 8–10 a.m.) and lasted 25 ± 6 min. The sessions took place in the children’s bedrooms, with only the experimenters present, ensuring a calm environment. Lighting was artificial and not systematically controlled.

### 2.6 EEG data analysis

#### 2.6.1 Preprocessing

From the raw signal, a first transformation was performed, using SPM8 software within a Matlab environment (Windows, Version R2018b, 9.5.0.944444) to obtain EDF files. Then, EEG and EOG signals were band-pass filtered between 0.25 and 30 Hz, and EMG signals between 10 and 100 Hz, in accordance with the recommendations of the American Academy of Sleep Medicine (AASM) (Troester et al., 2023). Mastoid electrodes were used as reference electrodes. No resampling was applied, and the original acquisition sampling rate of 500 Hz was maintained. Sleep epochs of 30 s were classified into five stages (Wakefulness, N1, N2, N3 and REM) according to the latest version of the manual of the AASM (Troester et al., 2023). This process was accomplished using the fMRI Artefact rejection and Sleep Scoring Matlab Toolbox (FASST) (Leclercq et al., 2011). Arousals were visually scored during sleep according to AASM criteria, defined as abrupt shifts in EEG frequency lasting at least 3 s and preceded by at least 10 s of stable sleep (Troester et al., 2023). Similarly, artifacts, such as movement or muscle-related activity, were visually identified and removed during both sleep and wakefulness. All arousal and artifact markings were independently verified by two experts. Finally, bad channels were manually excluded when more than 30 % of the recording contained noise or artifacts, which resulted in varying sample sizes across electrodes: Fz (N = 52); Cz (N = 51); Pz (N = 50); Oz (N = 49).

#### 2.6.2 Estimating aperiodic signal and periodic oscillations

Power spectra were first calculated separately for each participant, each electrode and each sleep stage. To do so, the continuous EEG signal was segmented in 6-s long windows overlapping by 50 %. Each window was labelled according to the sleep stage they occurred in and windows overlapping two different sleep stages were discarded. Then, each window was Hanning-tapered, before a fast Fourier transform was applied and the modulus of the complex numbers from this decomposition was squared to obtain the power spectrum. Within the same recording, power spectra of windows occurring in the same sleep stage were averaged together to obtain a single average power spectrum per participant, electrode and sleep stage. Subsequently, we applied the open-source, Python-based Fitting Oscillations and One-Over-F (FOOOF) toolbox (https://doi.org/10.1038/s41593-020–00744-x) to parameterize the average spectra through separation of the periodic and aperiodic components of the signal (Donoghue et al., 2020). Using this approach, power spectrums were treated as a linear combination of both aperiodic activity and oscillatory peaks with amplitudes that extend above the aperiodic signal (for a detailed overview of this approach, see (Donoghue et al., 2020, Cellier et al., 2021)). Using a model driven approach, the FOOOF algorithm is able to extract both periodic and aperiodic components within the overall power spectrum (Donoghue et al., 2020).

For the present study, the periodic components included the power and the centre of the frequency peak parameters. These parameters were extracted from the periodic signal for delta (0.5**—**4 Hz) on Fz in N3 sleep stage. This frequency range was selected based on literature and on visual inspection of the power spectra, which indicated clear peaks (i.e., ‘bumps’ in the power spectra) over-and-above the 1/f-like decay for most participants.

Regarding the aperiodic components, we extracted the slope across a broad frequency range between 0.25 and 30 Hz, similar to prior studies (Cellier et al., 2021), and as recommended in the FOOOF documentation. Fitting was performed using the ‘fixed’ aperiodic mode due to the absence of a clear ‘knee’ in the power spectrum when the output was visually inspected in log-log space (i.e., the signal was approximately linear across the specified frequency range). Spectral parameterization settings for the algorithm were: peak width limits = [1,12], maximum number of peaks = 8, peak threshold = [1.5**—**2], minimum peak height = 0.0. Uneven numbers of 6-s windows between sleep stages and participants resulted in average power spectra with different level of noise. Therefore, the peak threshold was adapted to the signal over noise ratio in each sleep stage and participant depending on the number of windows averaged together within the same session to obtain the average spectrum of the sleep stage. The peak threshold was selected as 3 if the number of spectra was below 100, as 2.5 if the number of spectra was between 100 and 500 and 2 is the number of spectra was above 500. The final FOOOF outputs were the aperiodic exponent and offset values, as well as the centre frequency, power, and bandwidth for the oscillatory component of the signal (see Fig. 1D).

#### 2.6.3 Sleep Spindle detection and analysis

We implemented the open-source and freely available Yet Another Spindle Algorithm (YASA) to detect sleep spindles during the N2 sleep stage using central (Cz) and parietal (Pz) electrodes as spindle’s spatial distribution shifts from frontal regions around 5 years old to central and parietal ones in late childhood and adolescence (Kwon et al., 2023). This algorithm is part of a broader sleep analysis package written in Python and available at https://github.com/raphaelvallat/yasa. YASA applies parameters tailored for typically developing children targeting spindle frequencies (11–16 Hz) and durations (0.5–3 s). As highlighted in the introduction, our study primarily investigated sleep spindle frequencies as a marker of brain development.

### 2.7 Statistical analysis

First, we assessed demographical differences in sleep parameters by calculating Pearson correlations between sleep parameters and age, stratified by gender (defined in the present study by Girls and Boys and not sex as biological attributes were not assessed). The sleep parameters examined included sleep stage durations, spindle features, and aperiodic components, as described above. Fisher tests were conducted to assess significant differences in correlation coefficients between independent groups. Additionally, we used a linear mixed-effects model to examine differences between Electrodes (within factor) in delta power during N3, while controlling for Gender and Age. This model included an Electrodes × Gender interaction, Age as a fixed subject-varying covariate, and a random intercept for subjects to account for repeated measures correlation of electrodes within subjects. The R formula for the model using the lme4 packages was: Delta Power ∼ Electrodes * Gender + Age + (1|Subject), where Subject refers to an individual participant identifier. Fixed effects were tested with a Type II ANOVA breakdown using F-tests, using Satterthwaite’s correction for denominator degrees-of-freedom (DDF). A significant Electrodes × Gender interaction was followed-up by pairwise *t*-tests of Electrodes within levels of Gender, again using Satterthwaite’s correction for DDF.

Next, a similar model was fitted to evaluate the overnight performance at the visual memory task. This model included a Session (Morning vs Evening) × Reward (Candies vs Vegetables) interaction, with Gender and Age as fixed subject-varying covariates, and a random intercept for subjects to account for repeated measures correlation. The R formula for the model using the lme4 packages was: Memory Score ∼ Session * Reward + Gender + Age + (1|Subject), where Subject refers to an individual participant identifier. Once again, fixed effects were tested with a Type II ANOVA breakdown using F-tests, and a significant Session × Reward effect followed by pairwise *t*-tests of Reward within Session levels.

All inferential tests were conducted at a reduced significance level of α = .005, to improve reproducibility of results and reduce the number of false positive tests (Benjamin et al., 2017). Under the reduced significance, we also considered as possible "trends" any effect where p was larger than .005 but smaller than .05. Finally, when comparing multiple electrodes, we still applied a Bonferroni correction to further safeguard against false positive results. As effect size, we calculated partial eta squared for F-tests and Cohen’s d for *t*-tests. Assumption diagnostics included checks for multicollinearity, influential cases (with Cook’s distance), non-normal residuals (with quantile-quantile plots), and heteroscedastic residuals (with partial dependence plots). Analyses were conducted using R (Version 2025.4.5.0) using the packages “lme4” and “lmerTest” for multilevel regression, packages “emmeans” and “effectsize” for follow-up comparisons and effect sizes.

## 3 Results

**Fig. 2:**
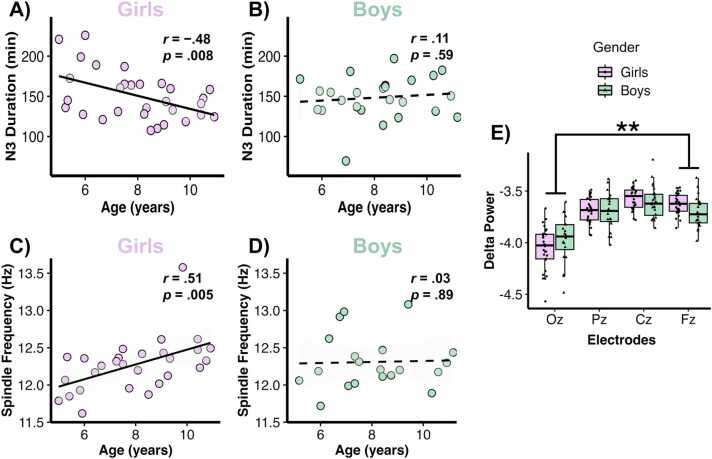
Evolution of sleep features with age and gender. (A) Pearson correlation between age and N3 duration for girls (N = 30). (B) Pearson correlation between age and N3 duration for boys (N = 25). (C) Pearson correlation between age and sleep spindle frequency on Pz for girls (N = 28). (D) Pearson correlation between age and sleep spindle frequency on Pz for boys (N = 20). (E) Boxplot of delta power for girls and boys on occipital to frontal midline electrodes. Error bars indicate interquartile range. The dashed line denotes p-values above .05. Asterisks represent the significance level (p) of Bonferroni post-hoc analyses: **p ≤ .01.

First, we looked at Pearson correlations between sleep parameters recorded by the PSG and age, computed for each gender. Overall, TST significantly decreased in girls with age (*r*(30) = -.53, *p* = .003, 95 % CI [-.75, −.21]) whereas no such association was observed in boys (*r*(24) = -.05, p = .82, 95 % CI [-.44,.36]). The difference between these correlations was significant (*z* = -1.86, *p* = .03, 95 % CI [-0.94,.03]) (see Supplementary Material, Table S1). Neither N1 (girls: *r*(30) = -.37, *p* = .05, 95 % CI [-.64, −.01] versus boys: *r*(25) = .25, p = .23, 95 % CI [-.16,.59]) nor N2 (girls: *r*(30) = .25, *p* = .18, 95 % CI [-.12,.56] versus boys: *r*(25) = -.18, p = .40, 95 % CI [-.54,.23]) did significantly change in duration across development in girls or boys. But we found that whereas girls showed a trend of decreasing N3 duration with age (*r*(30) = -.48, *p* = .008, 95 % CI [-.71, −.14]), boys did not (*r*(25) = .11, p = .59, 95 % CI [-.30,.49]; see Figs. 2A and 2B), with a statistically significant difference between correlations (*z* = -2.21, *p* = .014, 95 % CI of the mean difference [-1.03, −.06]). REM sleep displayed a similar pattern (girls: *r*(30) = -.53, *p* = .002, 95 % CI [-.75, −.21] versus boys: *r*(25) = -.30, p = .15, 95 % CI [-.62,.11]) although the difference between these correlations did not reach significance (*z* = -.98, *p* = .84, 95 % CI [-0.69,.22]) (see Figure S1A and S1B in Supplementary Material).

Next, we looked at features of sleep spindles in N2 sleep stage (on Cz and Pz), notably because of the well-established relationship between sleep spindles and memory consolidation processes (Chatburn et al., 2013, Fogel and Smith, 2011, Tamminen et al., 2010). After detection of sleep spindles (see Methods section), we computed the spindle density (spindles/minute of N2) and then extracted the mean duration, frequency, and relative power of the spindles. Both girls and boys showed a trend for higher spindle density on Pz with age (girls: *r*(28) = .43, *p* = .02, 95 % CI [.07,.69] and boys: *r*(20) = .56, *p* = .01, 95 % CI [.16;.80]), and had increased relative power (girls: *r*(28) = .52, *p* = .005, 95 % CI [.18,.75] and boys: *r*(20) = .58, *p* = .007, 95 % CI [.19;.81]) and longer spindles with increasing age (girls: *r*(28) = .47, *p* = .01, 95 % CI [.12,.72] and boys: *r*(20) = .46, *p* = .04, 95 % CI [.02;.75]). While spindle frequency significantly increased in girls with age (*r*(28) = .51, *p* = .005, 95 % CI [.17,.74]), boys did not show a significant association between these variables (*r*(20) = .03, *p* = .89, 95 % CI [-.42;.47]) (see Figs. 2C and 2D). Moreover, these correlations were suggested to be different from each other, with girls' correlation being higher than boys' (*z* = 1.69, *p* = .045, 95 % CI [-.07;.98]). Similar results were observed at Cz; however, for clarity of visual presentation, only the findings from Pz are shown below.

We then focused on one key periodic component of the EEG signal related to sleep homeostasis and synaptic downscaling, namely delta oscillatory activity (Ringli and Huber, 2011). We extracted delta power (0.5**—**4 Hz) computed across each electrode of the medial line (Oz, Pz, Cz and Fz) during N3 sleep. The Type II ANOVA breakdown of the fitted linear mixed model revealed no main effect of Gender (*F*(1, 50.57) = .05, *p* = .82) nor of Age (*F*(1, 50.04) = 2.10, *p* = .15), but a main effect of Electrodes (*F*(3, 141.53) = 45.02, *p* < .001, partial *η*^2^ = .49) and a significant interaction Gender*Electrodes (*F*(3, 141.23) = 5.83, *p* < .001, partial *η*^2^ = .11) (Fig. 2E). Bonferroni post-hoc tests revealed that delta power was higher in Fz compared to Cz (*t*(42 = −3.47, *p*_bonf_ =.004, *d* = −0.52) and to Oz (*t*(42) = 13.68, *p*_bonf_ < .001, *d* = 2.04), as well as in Cz compared to Oz (*t*(42) = 17.15, *p*_bonf_ < .001, *d* = 2.55) and in Pz compared to Oz (*t*(42) = 13.22, *p*_bonf_ < .001, *d* = 1.97). Regarding the interaction effect, we found that the difference in delta power between girls and boys was distinct across electrodes, particularly between Fz and Oz (*t(44)* = -2.98, *p* = .005, *d* = -.89). Specifically, boys exhibited higher delta power on Oz compared to girls, whereas on Fz, girls showed higher delta power than boys. The difference in Fz-Oz for boys (*M* = 0.24, *SD* = 0.23) differed from the difference Fz-Oz for girls (*M* = 0.42, *SD* = 0.19).

**Fig. 3:**
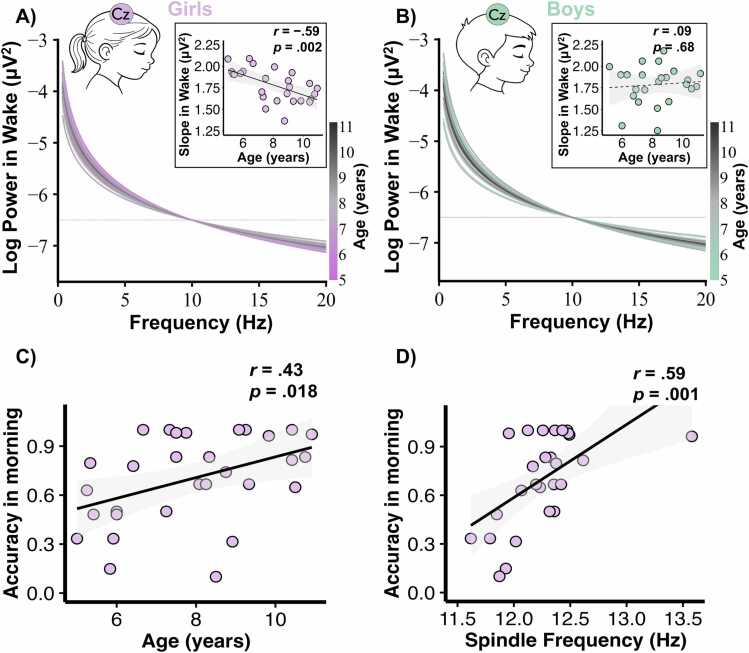
Correlations between brain activity during wakefulness, cognition, and age. (A) Slope of aperiodic component in wakefulness and Pearson correlation with age for girls (N = 26). (B) Slope of aperiodic component in wakefulness and Pearson correlation with age for boys (N = 24). (C) Pearson correlation between age and accuracy on the SART for girls (N = 30). (D) Pearson correlation between sleep spindle frequency on Pz and accuracy on the SART for girls (N = 27). The dashed line denotes p-values above .05.

Finally, we examined the aperiodic component (i.e., slope) of the signal on Cz during wakefulness and across sleep stages. As expected and consistent with previous findings, the slope increased across deeper sleep stages, reflecting the greater prevalence of slow waves and, consequently, enhanced cortical inhibition (Favaro et al., 2023) (see Figure S2 in Supplementary Material for the slopes in the different sleep stages). Beyond that aspect, our analysis revealed a significant decrease in the slope during wakefulness with age in girls (*r*(26) = -.59, *p* = .002; 95 % CI [-.79, −.26]), but not in boys (*r*(24) = .09, *p* = .68, 95 % CI [-.33,.47]) (see Figs. 3A and 3B). The difference between these two correlations was significant (*z* = -2.54, *p* = .006, 95 % CI [-1.12, −.15]). Regarding the slope across the other sleep stages, we only found a trend of displaying a decrease in the slope during in N1 with age in girls (*r*(26) = -.45, *p* = .02, 95 % CI [-.71, −.07]) whereas boys do not exhibit any effects (*r*(24) = .04, *p* = .86, 95 % CI [-.37,.44]). The difference between these correlations was significant (*z* = -1.74, *p* = .04, 95 % CI [-.97,.06]). Regarding the other sleep stages (i.e., N2, N3 and REM), we did not find any trend nor significant changes in the slope dependant of gender and age. Respectively for N2, N3 and REM, (see Figure S2 in Supplementary).

The second main aim of this study was to investigate potential bridges between sleep features and wakefulness performance, measured during cognitive tasks. To control for potential sleep inertia effects, we calculated the mean timing of task completion relative to children’s bedtimes and task onset relative to their wake-up times. On average, girls completed the evening tasks 1 h 35 min (SD = 58 min) before bedtime and began the morning tasks 1 h 45 min (SD = 53 min) after waking up. Similar results were observed in boys: evening tasks ended 1 h 37 min (SD = 46 min) before bedtime and morning tasks started 1 h 51 min (SD = 53 min) after waking up. Therefore, we analysed accuracy on the SART task performed in the morning, considering age and gender. Our findings indicated that girls showed a trend of increasing in accuracy with age (*r*(30) = .43, *p* = .018, 95 % CI [.08,.68]) (see Fig. 3C), whereas boys did not exhibit a similar pattern (*r*(25) = -.14, *p* = .51, 95 % CI [-.51,.27]), with a significant difference between these correlations (*z* = 2.09, *p* = .018, 95 % CI [.03, 1.02]). Given that age is associated with higher accuracy in girls and with changes in sleep spindle frequency, we computed the correlation between accuracy and sleep spindle frequency. We found that girls with faster sleep spindles had higher accuracy (*r*(27) = 0.59, *p* = .001, 95 % CI [.26,.79]) (see Fig. 3D), whereas boys showed an opposite trend (*r*(18) = -.43, *p* = .07; 95 % CI [-.75,.04]), with a significant difference between these correlations (*z* = 3.46, *p* = .0003, 95 % CI [.45, 1.40]).

Regarding the memory task, the Type II ANOVA breakdown of the fitted linear mixed model revealed no main effect of Gender (*F*(1, 58) = .002, *p* = .96) nor of Age (*F*(1, 50.04) = 2.10, *p* = .15), but a main effect of Session (*F*(1, 180) = 108.56, *p* < .001, partial *η*^2^ = .38), reflecting higher memory scores in the evening compared to the morning. Similarly, a main effect of Reward was found (*F*(1, 180) = 30.81, *p* < .001, partial *η*^2^ = .15) displaying higher memory scores for rewarded items (candies) compared to no rewarded ones (vegetables). The interaction Session*Reward was not significant (*F*(1, 180) = 1.37, *p* = .24). The raw data, along with analyses of PSG parameters, sleep diary entries, and cognitive task performance, all examined for age-related gender differences, are available in the supplementary material (see Supplementary Material, Table S1).

## 4 Discussion

Between the ages of 5 and 12, several sleep parameters changed linearly with age, but such a progressive evolution was mainly observed in girls. Indeed, girls showed a marked decreased in the duration of N3 sleep with age whereas, in boys, N3 duration appeared to remain stable. An earlier N3 shortening in girls during childhood is consistent with, and may explain why, Acebo et al. (Acebo et al., 1996) reported that girls aged 9–16 spent an average of 133 min in SWS, whereas boys spent 154 min. Beside the decrease in N3, our study revealed that age-related changes in the topography of delta power (posterior-to-anterior distribution), as observed by Kurth et al. (Kurth et al., 2010), also indicated a faster migration of SWA towards frontal regions in girls compared to boys. These changes in deep sleep physiology were paralleled by a progressive change in waking neuronal excitation/inhibition balance, characterized by a flatter slope of the aperiodic EEG component, indicating increased excitation with age. While Favaro et al. (Favaro et al., 2023) previously reported a decrease in the aperiodic slope from 2 to 17 years old during wakefulness (but not during sleep), we found that between the ages of 5 and 12, changes in the slopes were observed only in girls, not in boys. Reduced N3 duration, frontal SWA topography, and flattening of the aperiodic slope with age converge to suggest earlier onset of brain maturation processes in girls compared to boys during childhood (Stanyard et al., 2024).

Moreover, sleep spindles, which represent a defining oscillatory activity in NREM sleep, and which are known to index brain plasticity mechanisms, showed a linear increase in density, relative power and duration with age in both girls and boys. Sleep spindle frequency also significantly correlated with age, but only in girls. This result aligns with several longitudinal studies reporting an age-related acceleration of sleep spindles between 6 and 18 years old (Zhang et al., 2021, Hahn et al., 2019).

One additional main aim of this study was to explore potential links between age-related changes in neurophysiological sleep and/or wake states and cognitive performance. We did find that morning accuracy on the SART increased with age in girls (and not in boys), and with the sleep spindle frequency, which we established increased with age in the girls. Here too, our data extends the association between spindle frequency and cognitive performance in children, previously reported by Chatburn et al. (Chatburn et al., 2013), specifying that the link between faster sleep spindle frequencies and executive functions during childhood may be gender-dependent. Regarding overnight memory consolidation, we did not observe any effects related to specific sleep features, age, or gender after one night of sleep in our sample of 5- to 12-year-old children. The lower performance in the morning compared to the evening may be explained by the passage of time, as we did not include a daytime control group to account for this factor. The positive effect of reward aligns with existing literature, which shows that emotionally salient memories tend to be better retained than neutral ones (Massol et al., 2020). However, since the interaction was not statistically significant, we cannot conclude that sleep plays a specific role in emotional memory consolidation. This observation converges with the large variability of results obtained across studies regarding the role of sleep in memory processes in children compared to adults (Short et al., 2018). However, the lack of significant effects does not indicate that children do not benefit from sleep, as we do not have a control group with wakefulness condition. Instead, it could illustrate their capacity to consolidate information is not mainly dependent of the quantity of sleep. A similar interpretation has been suggested by Astill et al. (Astill et al., 2014) regarding sleep and motor skill learning (finger-tapping task), proposing that offline improvement can also be achieved across different brain states during wakefulness.

Altogether, our findings contribute to a detailed and nuanced description of gender-specific developmental patterns in sleep and brain maturation during childhood. However, these results should be interpreted with caution, as pubertal status was not measured or included in the analyses.

Indeed, distinct trajectories of brain changes as a function of gender between 5 and 12 years old may be linked to the influence of hormones and puberty starting earlier in girls compared to boys (Alotaibi, 2019, Barendse et al., 2018). A large body of evidence has highlighted the role of puberty in sleep and brain changes, particularly during adolescence (Lucien et al., 2021, Goddings et al., 2019, Vijayakumar et al., 2018). However, future longitudinal studies should further examine the onset of puberty in school-aged children and its impact on sleep physiology and cognition. Even when considering pubertal status, we do not claim that differing patterns of brain and cognitive development are fully explained by biological/genetic factors. Accordingly, parenting styles and social influence may also play a major role in sleep-related differences between girls and boys (Smith et al., 2019). Moreover, studies in adults have established that subjective and objective sleep measures are shaped by work-family responsibilities and gendered social expectations for their fulfillment (Forest et al., 2022).

These conclusions emphasize the importance of recognizing gender differences and their developmental trajectories, not to promote discriminatory responses from caretakers or teachers, but to enable an informed account of the emotional, cognitive, and sleep development of both genders. These differing trajectories observed during childhood may also provide valuable insights into the expression of gender-prevalent psychiatric disorders during adolescence and adulthood.

## 5 Limitations

This study has several limitations that warrant consideration. Firstly, we did not assess Tanner stages, as a measure of puberty in children. Evaluating Tanner stages involves physical examinations, usually performed by healthcare professionals, which was too intrusive and not adapted to our ecological, non-medical setting at home. In addition, previous research has shown that biological age correlates more closely with SWS maturation than Tanner stages (Feinberg et al., 2006). Nevertheless, pubertal status, whether assessed using Tanner staging, the Pubertal Development Scale, or biological measures and a larger age-range (i.e., including older boys in the sample) should be considered to further explore potential links with hormonal changes (which was not done here). Secondly, we used a small number of EEG electrodes, which limits the precision of topographical information obtained from EEG data. However, following recent recommendations in developmental cognitive neuroscience EEG research, using a small number of freely placed electrodes is preferred for home recordings in children, as it allows for faster application, greater comfort, better inclusion across different head shapes and hair types, and improved overall feasibility and accessibility (Gaudette et al., 2025). A third limitation concerns the recording of only a single night of sleep, without an adaptation night being performed. The lack of an adaptation night may have induced a first-night effect, which is known to alter sleep quality and quantity, in adults but also in children (Ding et al., 2022). However, a meta-analysis revealed that (1) there were no significant gender differences and (2) some sleep features, such as SWS, were not affected by the first-night effect (Ding et al., 2022). Additionally, the first-night effect is typically observed in laboratory settings, where environmental sleep conditions differ substantially from habitual sleep environments. In the present study, all recordings were conducted in the children’s homes, thereby potentially limiting such a “laboratory effect”. Therefore, it is highly unlikely that the first-night effect accounts for the gender differences observed in the present study. These experimental choices were made to allow for ecological recordings at the children's home. On the one hand, home-based recordings have been shown to be less affected by the general “laboratory effect,” with studies reporting, for instance, longer sleep duration and better sleep efficiency at home (Marcus et al., 2014, Withers et al., 2022). Therefore, home-based recordings offer a unique opportunity to capture children’s natural sleep habits in an ecologically valid way, which is particularly valuable for research in pediatric populations. They also provide a faster, more affordable, yet reliable tool for clinical sleep screening (Withers et al., 2022). On the other hand, several environmental variables are not controlled for in a typical home setting and could have impacted both cognitive task performance and sleep. These include variations in bedroom noise (Caddick et al., 2018), light exposure (Hartstein et al., 2022), room temperature (Caddick et al., 2018), bedtime routines, and the use of electronic devices before sleep (Hartstein et al., 2024). Similarly, we did not distinguish between slow (<13 Hz) and fast (>13 Hz) sleep spindles, where changes in slow sleep spindles might be more relevant for the maturation of frontal brain networks and cognitive functions (Hahn et al., 2019). Indeed, given our limited number of electrodes and the absence of spindle frequency norms as a function of biological gender in children, we used the standard frequency of sleep spindles, i.e., 11–16 Hz, for the detection of sleep spindles. A sixth potential limitation pertains to the assessment of cognitive performance. Here, we looked at changes in performance relative to changes in sleep due to development (i.e., age), but not at the absolute impact of one night of sleep (versus no sleep). The latter analysis was beyond the scope of the present study and would require a wake or sleep deprivation control group. Another limitation is the lack of information on general parenting styles, family history of mental disorders, socioeconomic status, peer relations and ethnicity. These factors have been shown in previous research to influence children's sleep patterns and overall health (Bøe et al., 2012). Addressing these limitations and using longitudinal measurements are needed to provide further support to our initial description of the developmental interplay between sleep, gender and cognitive functions in children.

## 6 Conclusion

When compared to boys, school-aged girls spent less time in the deepest sleep stage with age, and showed a more frontal topography of delta power, as well as faster sleep spindles. They also exhibited more excitatory brain activity during wakefulness, and higher cognitive performance by the age of 12 years old. Please note that inference about causal relationships between these variables remains beyond the scope of the present cross-sectional, correlational study. Altogether, our findings highlight the need for further research to elucidate gender differences, as well as the likely influence of environmental factors, in sleep and cognitive developmental trajectories in healthy children. Further ecological assessments of differential sleep patterns, as performed here, could provide valuable early biomarkers for the development of cognitive and mental health later in life and their potential perturbations.

## Additional Information

None

## Author contributions

K.M. wrote the manuscript. V.S., F.J., N.F., H.R.M., J.C. and S.S. designed the research. V.S., F.J., N.F., H.R.M. and J.C. acquired the data. K.M., G.L. and V.S. analysed the data. All authors edited, reviewed, and have approved the final manuscript.

## CRediT authorship contribution statement

**Guillaume Legendre:** Writing – review & editing, Validation, Supervision, Software, Resources, Methodology, Formal analysis. **Kevin Mammeri:** Writing – review & editing, Writing – original draft, Visualization, Software, Project administration, Formal analysis. **Virginie Sterpenich:** Writing – review & editing, Visualization, Validation, Supervision, Resources, Project administration, Methodology, Investigation, Funding acquisition, Data curation, Conceptualization. **Joanny Combey:** Methodology, Investigation, Data curation, Conceptualization. **Helene Ruppen-Maret:** Methodology, Investigation, Data curation, Conceptualization. **Nathalie Fernandez:** Methodology, Investigation, Data curation, Conceptualization. **Fiona Journal:** Methodology, Investigation, Data curation, Conceptualization. **Sophie Schwartz:** Writing – review & editing, Supervision, Resources, Project administration, Funding acquisition, Conceptualization.

## Funding statement

This work was supported by the Swiss National Science Foundation (grant number: 320030_182589), by the Gertrude von Meissner foundation and was hosted by the University of Geneva.

## Ethics approval statement

Approved by the Geneva University Research Ethics Commission (CUREG) on June 4, 2018.

## Patient consent statement

Children’ informed consents were obtained from a parent and/or legal guardian.

## Permission to reproduce material from other sources

Non applicable

## Clinical trial registration

No registration

## Data Statement

The data that support the findings of this study are available on request from the corresponding author.

## Declaration of Competing Interest

The authors have nothing to declare.

## Data Availability

Data will be made available on request.
