# Supplementary material for: Age and gender-related neurophysiological changes in sleep and wake states during childhood
Source: Dev Cogn Neurosci. 2026 Jan 21;78:101681. doi: 10.1016/j.dcn.2026.101681 (PMC12861257; doi:10.1016/j.dcn.2026.101681)
Supplement: Supplementary file 1 — Supplementary material [file mmc1.docx]

**Supplementary Material**

Age and gender-related neurophysiological changes in sleep and wake states during childhood

Kevin Mammeri^1,2,*^, Guillaume Legendre^1,2^, Fiona Journal^1,2^, Nathalie Fernandez^1,2^, Helene Ruppen-Maret^1,2^, Joanny Combey^1,2^, Sophie Schwartz^1,2^ & Virginie Sterpenich^1,2,*^

^1^Department of Basic Neurosciences, University of Geneva, Switzerland

^2^Swiss Center for Affective Science, Geneva, Switzerland,

*Corresponding authors. Kevin Mammeri, Campus Biotech, Chemin des Mines, 9, 1202 Geneva, Switzerland. Email: Kevin.Mammeri@unige.ch; Virginie Sterpenich. Email: Virginie.Sterpenich@unige.ch.

**Table S1.** Demographic comparisons and age correlations by gender.

|  |  | Boys | | | Girls | | |  |  |  |  |
| --- | --- | --- | --- | --- | --- | --- | --- | --- | --- | --- | --- |
|  |  | n | M (SD) | Correlation with Age (r (p)) | n | M (SD) | Correlation with Age (r (p)) | Mean Difference | t | df | p |
|  | Age (years) | 30 | 8.12 (1.66) |  | 31 | 7.92 (1.81) |  | .20 | .45 | 59 | .66 |
| Sleep Diary  (7 days) |  |  |  |  |  |  |  |  |  |  |  |
|  | TST (h) | 30 | 10.06 (.51) | **-.61 (.001)** | 31 | 10.20 (.72) | **-.60 (.001)** | -.14 | -.86 | 59 | .39 |
|  | Bedtime | 30 | 21.15 (.63) | .38 (.04)* | 31 | 21.10 (.56) | .25 (.18) | .05 | .32 | 59 | .75 |
|  | Waking-up time | 30 | 7.48 (.30) | .04 (.84) | 31 | 7.58 (.60) | -.46 (.009)* | -.19 | -.82 | 59 | .42 |
|  | Sleep Quality  (Max = 6; Higher Scores = Better Quality) | 29 | 5.09 (.75) | 0.0001 (1) | 31 | 5.00 (.69) | -.39 (.03)* | .08 | .45 | 58 | .65 |
|  | Mood (Max = 5; Higher Scores = More Positive) | 30 | 4.35 (.47) | -.17 (.38) | 31 | 4.60 (.40) | -.10 (.61) | -.24 | -2.18 | 59 | .03* |
| EEG (1 night) |  |  |  |  |  |  |  |  |  |  |  |
|  | TST (h) | 24 | 8.66 (.91) | -.05 (.82) | 30 | 8.79 (.75) | **-.53 (.003)** | -.13 | -.58 | 52 | .56 |
|  | N1 (min) | 25 | 37.0 (20.11) | .25 (.23) | 30 | 33.9 (19.23) | -.37 (.05) | 3.10 | .58 | 53 | .56 |
|  | N2 (min) | 25 | 223 (38.42) | -.18 (.40) | 30 | 229 (36.96) | .25 (.18) | -6.26 | -.62 | 53 | .54 |
|  | SE (%) | 24 | 91.8 (.08) | .13 (.56) | 30 | 92.4 (.06) | .17 (.36) | -.01 | -.33 | 52 | .74 |
|  | WASO | 24 | 2.8 (1.08) | .26 (.22) | 30 | 2.28 (.94) | -.004 (.98) | .52 | 1.91 | 52 | .06 |
|  |  |  |  |  |  |  |  |  |  |  |  |
| SART |  |  |  |  |  |  |  |  |  |  |  |
|  | Accuracy (Evening) | 26 | .76 (.21) | .10 (.64) | 29 | .76 (.21) | -.03 (.88) | .004 | .07 | 53 | .95 |
|  | Accuracy (Morning) | 25 | .84 (.16) | -.14 (.51) | 30 | .70 (.27) | .43 (.02)* | .14 | 2.28 | 53 | .03* |
|  | RT (ms) (Evening) | 26 | 475 (120.08) | **-.67 (.001)** | 30 | 487 (157.67) | **-.68 (.001)** | -12.20 | -.32 | 54 | .75 |
|  | RT (ms) (Morning) | 25 | 395 (78.04) | -.53 (.006)* | 30 | 453 (133.86) | **-.77 (.001)** | -58.22 | -1.92 | 53 | .06 |
| Memory Task (Scores) |  |  |  |  |  |  |  |  |  |  |  |
|  | Rewarded Memory (Evening) | 30 | 3.43 (.60) | .23 (.23) | 31 | 3.37 (.81) | .38 (.04)* | .06 | .34 | 59 | .73 |
|  | No Rewarded Memory (Evening) | 30 | 2.87 (.84) | .11 (.55) | 31 | 2.81 (.84) | -.18 (.34) | .06 | .28 | 59 | .78 |
|  | Rewarded Memory (Morning) | 30 | 2.37 (.79) | .17 (.37) | 31 | 2.48 (.93) | -.02 (.91) | -.12 | -.53 | 59 | .60 |
|  | No Rewarded Memory (Morning) | 30 | 2.08 (.80) | -.02 (.94) | 31 | 2.03 (.81) | .03 (.87) | .05 | .25 | 59 | .81 |


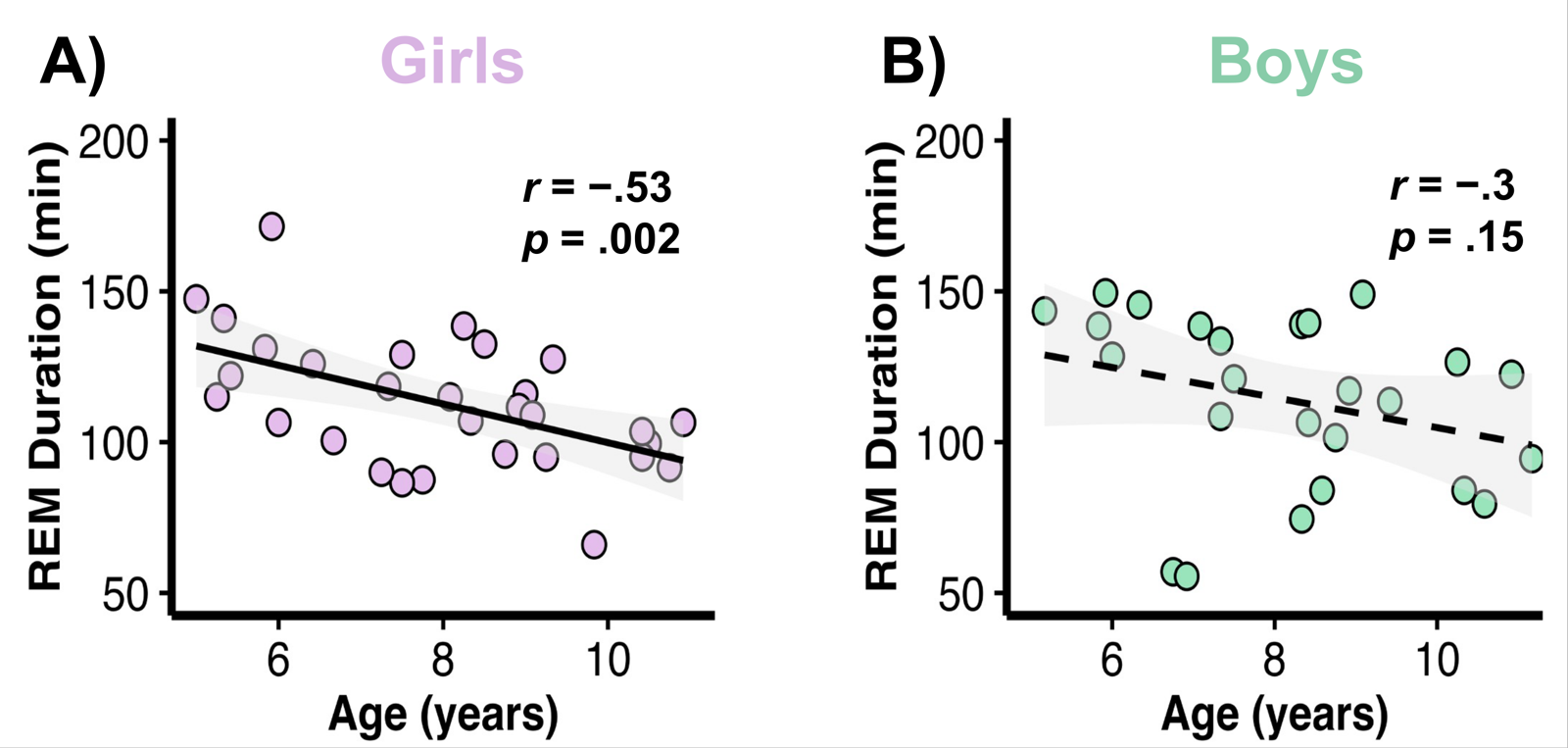
**Figure S1**.


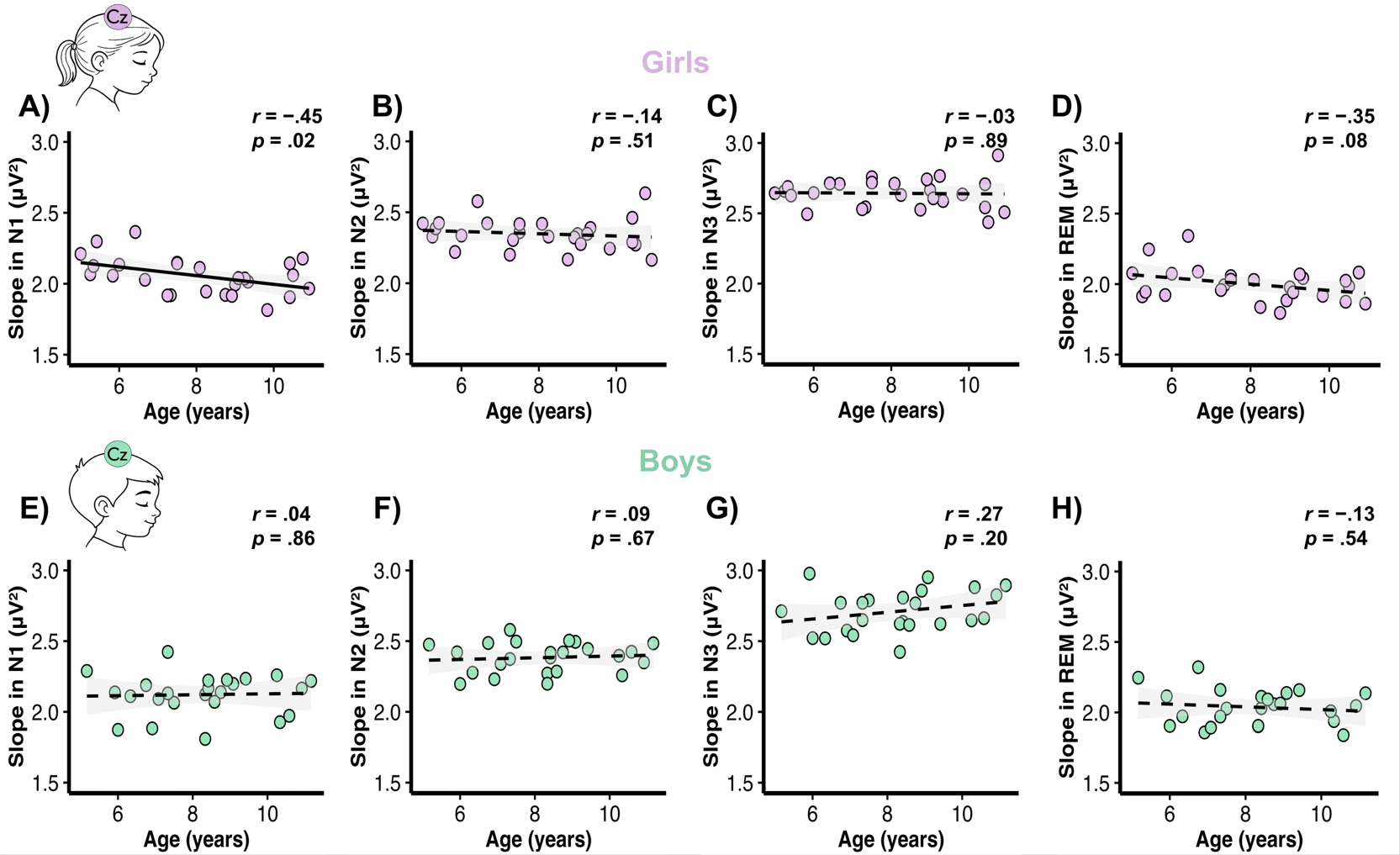
**Figure S2**.

**Table and Figure Captions**

**Table S1.** Demographic comparisons between girls and boys, with group comparisons (t-tests) and correlations with age (Pearson’s r). Bold represents significant p-values (p<.005). Asteriks represent a trend (.005<p<.05).

**Figure S1**. Evolution of sleep stages duration with age and sex. (A) The Pearson correlation between age and REM duration for girls (N = 30). (B) The Pearson correlation between age and REM duration for boys
(N = 25). (D). The dashed line denotes p-values above .05.

**Figure S2.** Correlations between slopes and age between sexes through all sleep stages. (A) Pearson correlation of slopes in N1 according to age for girls (N = 26). (B) Pearson correlation of slopes in N2 according to age for girls (N = 26). (C) Pearson correlation of slopes in N3 according to age for girls (N = 26). (D). Pearson correlation of slopes in REM according to age for girls (N = 26). (E) Pearson correlation of slopes in N1 according to age for boys (N = 24). (F) Pearson correlation of slopes in N2 according to age for boys (N = 24). (G) Pearson correlation of slopes in N3 according to age for boys (N = 24). (H) Pearson correlation of slopes in REM according to age for boys (N = 24). The dashed line denotes p-values above .05.
